# Heparin-induced Anaphylaxis shock

**DOI:** 10.5339/qmj.2023.sqac.17

**Published:** 2023-11-26

**Authors:** Anas Mohamed, Amro Abdelrahman, Khalid Y Fadul, Mohamed Elgassim

**Affiliations:** ^1^Hamad Medical Corporation; Medical Education Doha, Qatar Email: Amohamed47@hamad.qa

## Abstract

**Introduction:** Anaphylaxis is a fatal condition that can be easily managed if discovered early. Only a few examples of anaphylaxis-like reactions caused by heparin have been documented, and immediate-type hypersensitivity reactions to heparin are extremely uncommon.

**Case Presentation:** We report a case of a 53-yearold gentleman known to have an End Stage Renal Disease (ESRD) on Hemodialysis for two years, who went to the dialysis facility in his usual state of health. After two hours of dialysis, a new lock of taurolidine/heparin was installed; one minute later, the patient started to vomit and became restless, blood pressure dropped to 60/47 mmHg, and urticarial hives and a reddish rash developed on his skin, covering his trunk and limbs. He was immediately given three doses of epinephrine intramuscularly, which he did not respond to. Therefore, an epinephrine infusion was started. IV hydrocortisone and diphenhydramine were given for symptomatic relief. The patient was shifted to the emergency department, where he became vitally stable and returned to baseline. Heparininduced anaphylaxis was assumed based on the quick response to the above medications.

**Conclusion:** This case can be added to the growing literature regarding this rare reaction, and more studies should be done to understand the nature of the reaction better. We recommend that the healthcare team becomes vigilant of heparin as a possible cause of anaphylaxis.

